# Delayed Diagnosis of Primary Ciliary Dyskinesia in Low-Middle-Income Countries: The Clinical Value of Nasal Nitric Oxide

**DOI:** 10.7759/cureus.71888

**Published:** 2024-10-19

**Authors:** Diana Ortiz-Farìas, Alma Rodríguez-Guzmán, Arturo Cortes-Telles, Esperanza Figueroa-Hurtado

**Affiliations:** 1 Clínica de Enfermedades Respiratorias, Hospital Regional de Alta Especialidad de la Península de Yucatán-IMSS Bienestar, Mérida, MEX

**Keywords:** chronic cough, dyspnea, nasal nitric oxide, non-cystic fibrosis bronchiectasis, primary ciliary dyskinesia

## Abstract

Primary ciliary dyskinesia (PCD) is a rare lung disease that causes chronic oto-sino-pulmonary disease with irreversible lung damage. Several diagnostic methods exist, but electron microscopy (EM) is the most accurate tool, as it visualizes alterations in the axonemal ultrastructure; however, some patients may present a normal ciliary structure. Therefore, other diagnostic methods have been promoted, such as genetic studies or immunofluorescence of specific markers; nonetheless, they are not very accessible and expensive and even present a high level of false negatives. The quantification of nasal nitric oxide (nNO) has been a well-known tool for decades for the screening of this pathology, and recent studies have highlighted its high predictive value in the diagnosis of PCD, as it is a rapid tool in its processing, execution, and accessibility, especially in countries with limited health resources. We present the case of a patient with respiratory symptoms since childhood and extensive lung damage (cystic bronchiectasis); due to lack of access to EM or immunofluorescence, determinations of nNO were performed and found to be compatible with PCD.

## Introduction

Primary ciliary dyskinesia (PCD) is a rare congenital disease with several genetic mutations of the cilia that impair ciliary movement and is genetically heterogeneous, with autosomal-recessive character; however, there are also autosomal-dominant and X-linked profiles. Mucociliary clearance is one of the defense mechanisms of the respiratory tract. Cilia are fundamental structures present throughout the epithelium of the upper and lower respiratory tract; they are organelles with rhythmic and coordinated movements to move different substances and bacteria trapped on the mucosal surface. There are two general classes of cilia: motile and immotile. Their core structure is the axoneme, composed of nine doublets of microtubules that extend longitudinally and are anchored to the cell by a protein structure known as the basal body [1-3].

There are no clear diagnostic criteria; however, the Genetic Disorders of Mucociliary Clearance Consortium (GDMCC) proposes the following three groups: (a) zero to one month of age: situs inversus totalis and unexplained respiratory distress at birth, plus at least one of the following: altered ciliary ultrastructure, bi-allelic mutations in genes associated with PCD, or abnormalities of ciliary function; (b) one month to five years: two or more main clinical criteria (unexplained neonatal respiratory distress, organic laterality defects, cough with chronic expectoration, bronchiectasis, chronic nasal congestion or pansinusitis) plus at least one of the following: altered ciliary ultrastructure, bi-allelic mutations in a gene associated with PCD, or abnormalities of ciliary function; and (c) five to 18 years and adults: two or more major clinical criteria plus at least one of the following: low nasal nitric oxide (nNO), altered ciliary ultrastructure, bi-allelic mutations in a gene associated with PCD, or abnormalities of ciliary function [4,5].

Evaluation of axonemal ultrastructure by electron microscopy (EM) has become the gold standard, achieving the diagnosis in up to 70% of patients with PCD [5]. However, 30% of patients with a diagnosis confirmed by genetic studies may have a normal ultrastructure; in these scenarios, it is advisable to incorporate immunofluorescent staining for specific markers to accurately identify abnormal axonemal ultrastructure [6-8].

Different factors limit the use of EM in emerging low-middle-income countries (L-MIC), among which are lack of infrastructure, high costs, long time to obtain the results, and the high presence of unreliable results due to inadequate sampling. However, other diagnostic methods provide certainty for the identification of this pathology, especially the quantification of nNO, which has been reported as an additional element in the diagnosis of cases with PCD, and nNO levels <105 ppb reach a positive predictive value of 89% for a correct diagnosis The value of using nNO as a screening tool for PCD is clear, but there are a number of other conditions in which reduced nNO levels occur including cystic fibrosis, although usually not as low as in PCD [9].

We present the case of a woman in the fourth decade of life treated for recurrent respiratory symptoms and nNO measurement compatible with PCD.

## Case presentation

The patient is a 36-year-old female, native and resident of Yucatán, Mexico, with no history of smoking, drug addiction, and exposure to biomass. She has a history of allergy to ciprofloxacin and multiple respiratory infections since childhood and no family history of chronic respiratory disease. 

From the age of 16, the patient presented with progressive dyspnea up to mMRC4, which caused exercise limitation, cough attacks with expectoration, nasal congestion, and multiple episodes of sinusitis, in addition to difficulty in getting pregnant despite not using any planning method and having an active sexual life with her partner (sterility). At 18 years old, she was diagnosed with idiopathic cystic bronchiectasis and was treated with indacaterol/glycopyrronium and nebulized amikacin. However, she had multiple episodes of exacerbations that required hospital admissions, the most recent events being admitted to our hospital in October and December 2023, which caused a drop in functional class and the need for supplemental oxygen at home.

**Figure 1:**
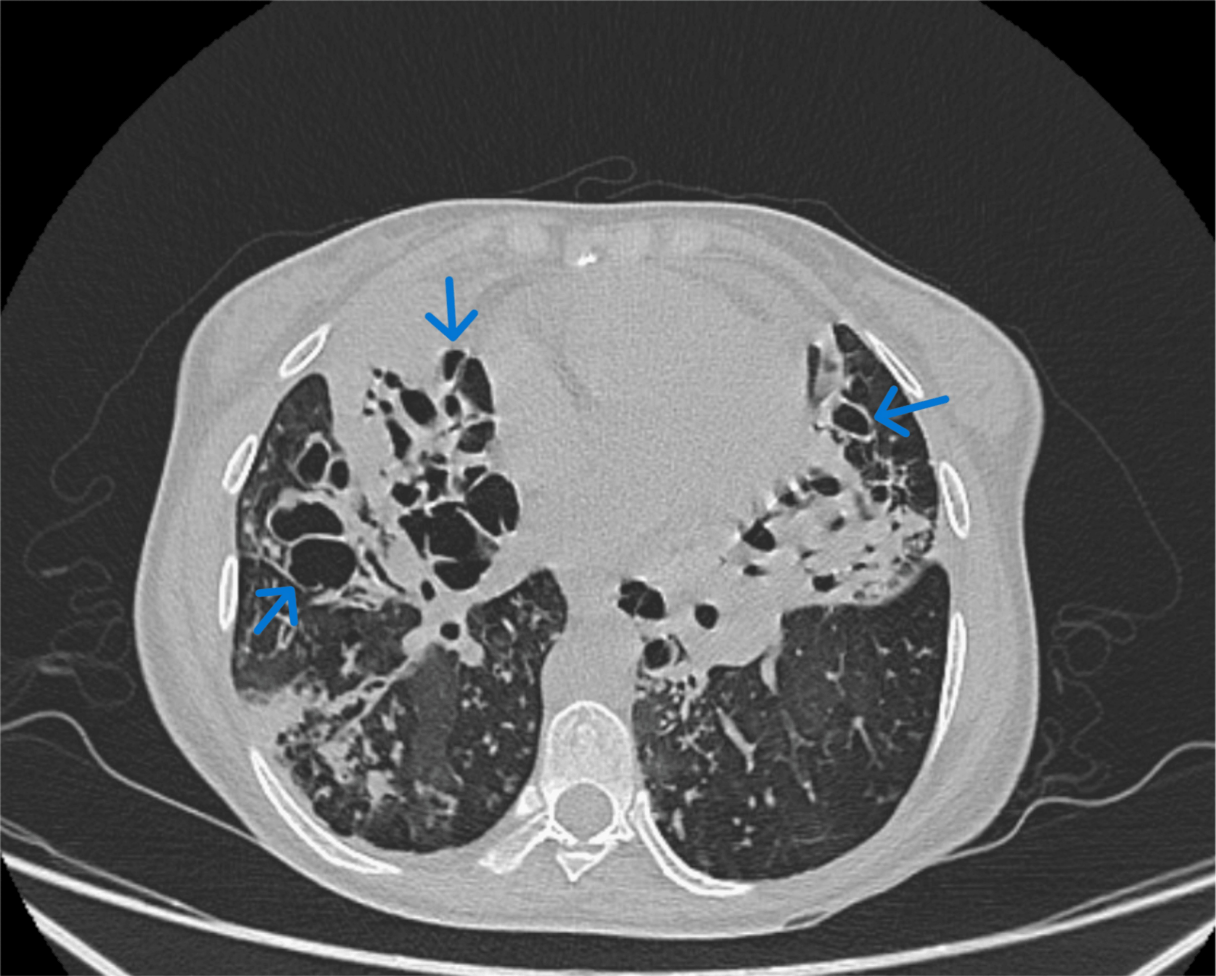
Cystic bronchiectasis CT scan of the lung with cystic bronchiectasis distorting architecture

**Figure 2:**
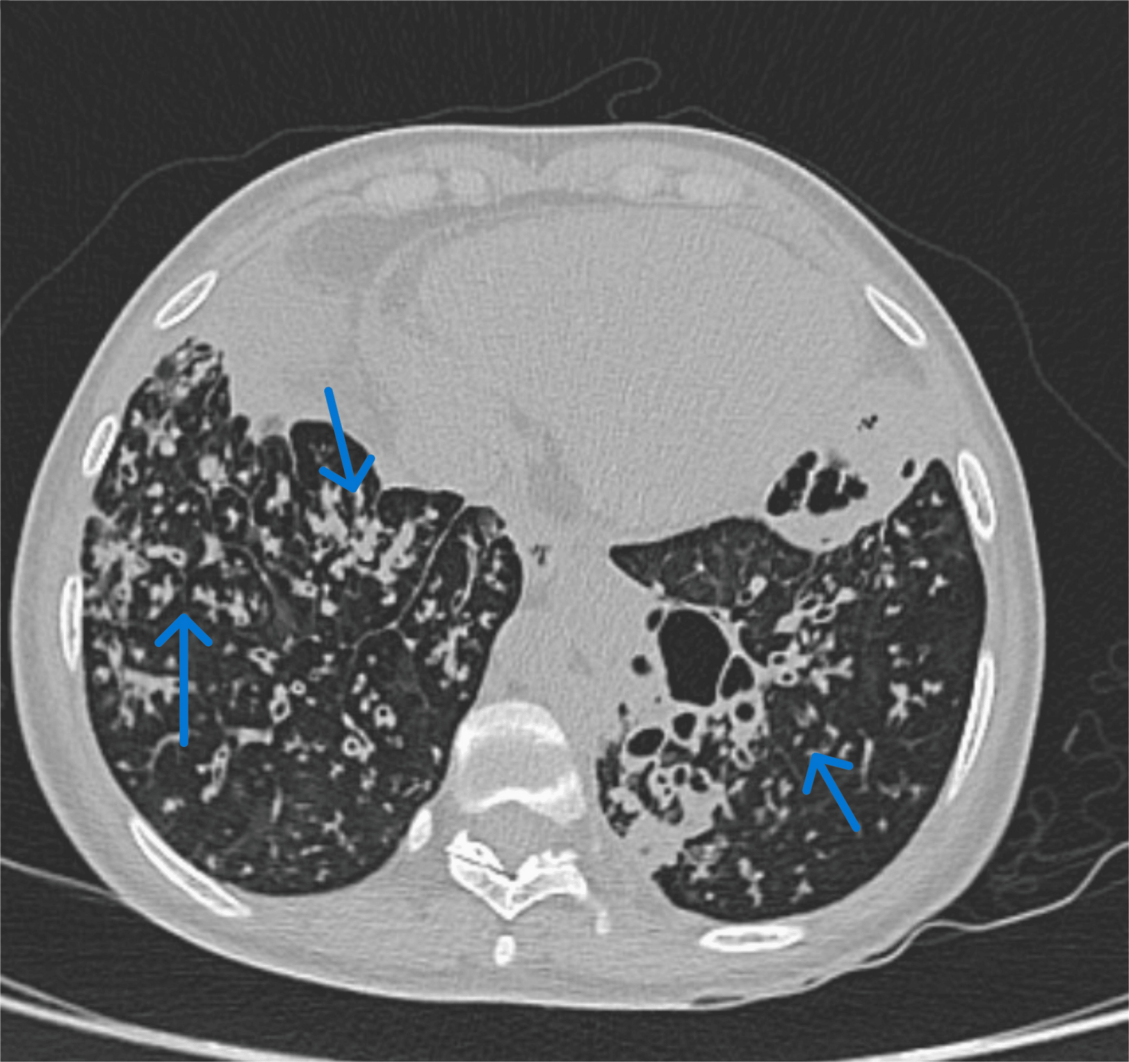
Tree-in-bud pattern Nodular lesions of centrilobular distribution, tree-in-bud pattern

In October 2023, a high-resolution chest tomography (HRCT) scan was requested, showing multiple lung segments with cystic bronchiectasis with random distribution and tree-in-bud pattern (Figure 1 and Figure 2).

Expectoration culture documented colonization with *Pseudomonas aeruginosa*. The etiological search for bronchiectasis was complemented with an immunological panel and complement (both with normal results) and multiple negative smear tests including negative GeneXpert and polymerase chain reaction (PCR) for tuberculosis. Due to respiratory distress and poor functional reserve based on HRCT findings, bronchoscopy is not performed. However, given the clinical suspicion of PCD, a two-time quantification (two measurements) of nNO was requested, reporting an average of 15 PPB or 4.3 nL/min confirming the clinical diagnosis.

## Discussion

PCD is a rare disease with a prevalence of one in 10,000-20,000 live newborns in L-MIC, although some reports indicate that it can reach a frequency of 5% in non-Hispanic children with recurrent respiratory infections and rates are often underestimated because many patients remain undiagnosed. In Mexico, information on the incidence and prevalence of the disease is unknown; we are only limited to reports of pediatric cases, emphasizing that in L-MIC, programs aligned to international standards for timely diagnosis, follow-up, and treatment do not exist [2-3,10-11].

Impaired ciliary function consequently contributes to chronic respiratory diseases including rhinitis, sinusitis, otitis media, suppurative lung disease, and bronchiectasis predominantly in the middle, lingual, or lower lobe. The presence of situs inversus and heterotaxia may be up to 50% of patients with or without congenital heart disease. Other findings that may be present are fertility disorders all due to sperm dysmotility and fallopian tube dysfunction [3,6].

Quantification of nNO levels is a more powerful tool that has proven to be useful not only in the screening but also in the diagnosis of PCD. A cut-off value of nNO less than 77 nL/min is closely associated with confirmed cases of PDC expressing ciliary axonemal defects or mutations in DNAH11 with a sensitivity of 98% and a specificity of 99% in children over five years of age with a compatible clinical presentation. In addition, the quantification of nNO levels can be considered as an alternative tool in cases with high suspicion of PDC [5,9,11,12]. The case of our patient is compatible with multiple of the characteristics described (repeated infections since childhood, history of sinusitis, sterility); by tomography, she had cystic bronchiectasis, in addition to having low levels of nNO (4.3 nL/min). 

Within the airway, nitric oxide is produced by numerous cell types, including epithelial cells, endothelial cells, fibroblasts, activated macrophages, nerve cells, and airway and vascular smooth muscle cells. With regard to ciliary function, in vitro studies of animal and human airway ciliated epithelium suggest that the induction of nitric oxide synthase (NOS) and nitric oxide production increases ciliary beat frequency; additionally, the production of nitric oxide within the airway is protective against infection and has bacteriostatic and bactericidal activity. The mechanism by which nNO is reduced in PCD has yet to be elucidated. The results have been conflicting, but cultured epithelial cells from PCD patients seem to produce equivalent amounts of nitric oxide, at baseline, as cells from non-PCD patients [7,9,13].

In Mexico, information on this disease is limited because there are few centers with adequate resources for diagnosis and comprehensive treatment; therefore, nNO quantification can be considered a routine test in the diagnostic approach of PCD, since it is more accessible to low-income countries.

## Conclusions

Recognizing PCD is difficult as the symptoms seen in PCD often overlap with those of other chronic respiratory diseases. In low-income countries, it is important to identify early symptoms associated with the disease including neonatal respiratory distress despite term birth, year-round productive (often moist) cough starting before six months of age, year-round non-seasonal nasal congestion starting before six months of age, and any organ laterality defects (including situs inversus totalis or situs ambiguus) to perform diagnostic tests that are inexpensive and easy to replicate in order to initiate treatments in early stages and improve the survival of our patients. There is no gold standard diagnostic test for PCD as each modality lacks sufficient diagnostic sensitivity and specificity. The nNO is a non-invasive, accurate, and lower-cost test compared to EM. When nNO is performed with a standardized protocol in the appropriate clinical context, values ≤77 nL/min are 96.3% sensitive and 96.4% specific for PCD. In low-income countries where access to genetic and microscopic diagnostic tests is very difficult, nNO testing is a useful first-line test to increase or decrease the suspicion of disease.
